# Patient perspectives on molecular tumor profiling: “W*hy wouldn’t you?”*

**DOI:** 10.1186/s12885-019-5920-x

**Published:** 2019-07-31

**Authors:** Megan C. Best, Nicole Bartley, Chris Jacobs, Ilona Juraskova, David Goldstein, Ainsley J. Newson, Jacqueline Savard, Bettina Meiser, Mandy Ballinger, Christine Napier, David Thomas, Barbara Biesecker, Phyllis Butow, Members of the PiGeOn Project, Katherine Tucker, Timothy Schlub, Richard Vines, Kate Vines, Judy Kirk, Mary-Anne Young

**Affiliations:** 1 University of Sydney, School of Psychology, Psycho-Oncology Co-operative Research Group (PoCoG), Level 6 North, Lifehouse C39Z, Sydney NSW, Sydney, 2006 Australia; 2 University of Technology Sydney, Graduate School of Health, Broadway NSW, Sydney, 2007 Australia; 3 Prince of Wales Clinical School, University of NSW, High Street, Kensington, NSW 2032 Australia; 4 Faculty of Medicine and Health, Sydney Health Ethics, University of Sydney, 92/94 Parramatta Road, Camperdown, NSW 2050 Australia; 5 School of Medicine, Deakin University, 1 Gheringhap Street, Geelong, Victoria 3220 Australia; 6 Psychosocial Research Group, Prince of Wales Clinical School, University of NSW, HighStreet, Kensington, NSW 2032 Australia; 7 Cancer Division, Garvan Institute of Medical Research, 384 Victoria street, Darlinghurst, NSW 2010 Australia; 8 RTI International, 701 13th St, NW #750, Washington, DC 20005 USA

**Keywords:** Cancer, Qualitative, Molecular tumor profiling, Genomic, Patient attitudes, Psychosocial, Interviews, Motivation

## Abstract

**Aim:**

This study explored the attitudes of patients with advanced cancer towards MTP and return of results, prior to undergoing genomic testing within a research program.

**Methods:**

Participants were recruited as part of the longitudinal PiGeOn (Psychosocial Issues in Genomics in Oncology) study involving patients with advanced/metastatic solid cancer who had exhausted therapeutic options and who were offered MTP in order to identify cognate therapies. Twenty patients, selected by purposive sampling, were interviewed around the time they gave consent to MTP. Interviews were audio recorded, transcribed and analysed using thematic analysis. Themes identified in the transcripts were cross-validated via qualitative responses to the PiGeOn study survey (*n* = 569; 63%).

**Results:**

All interviewed participants gave consent to MTP without reservation. Three themes were identified and further supported via the survey responses: (1) *Obvious agreement to participate*, primarily because of desire for new treatments and altruism. (2) The black box – while participant knowledge of genomics was generally poor, faith in their oncologists and the scientific process encouraged them to proceed with testing; and (3) *Survival is the priority* – receiving treatment to prolong life was the priority for all participants, and other issues such as identification of a germline variant were generally seen as ancillary.

**Conclusion:**

Having advanced cancer seemed to abrogate any potential concerns about MTP. Participants valued the research for varied reasons, but this was secondary to their priority to survive. While no negative attitudes toward MTP emerged, limitations in understanding of genomics were evident.

**Electronic supplementary material:**

The online version of this article (10.1186/s12885-019-5920-x) contains supplementary material, which is available to authorized users.

## Background

Molecular tumor profiling (MTP) is a form of genomic testing which aims to link molecular targets in tumors to cognate therapies. This allows identification of personalized treatment for cancer, such as potentially identifying new agents for treating a particular cancer and/or increasing efficacy of therapy while reducing unnecessary side-effects [1]. MTP has entered clinical practice, driven by improved technology, patient demand and the need for more effective cancer treatments [2]. However, it is not known how patients will respond to the potential challenges of such testing. A recent systematic review [3] found that most of the scant research in this area has been hypothetical. The little that is known suggests that patients, despite misunderstandings about genomics, are aware of the benefits of tumor testing (i.e. informing treatment) and are willing to undergo testing [4, 5]. However, patients have also reported some concerns, including worries about psychological harm related to intrusive thoughts due to unwanted knowledge. Potential privacy and insurance and/or employment discrimination are also of concern, though participant confusion with germline findings has also been noted [4, 6].

MTP involves testing tumor samples for identification of any genomic or molecular alterations to guide treatment choices. These alterations can affect treatment (clinically actionable), not affect treatment (non-actionable) or be of uncertain significance. There is a chance that germline variants, which have implications for biological relatives, may be identified [7]. In this case confirmatory testing is warranted. Miller and colleagues found that in a sample of patients with advanced cancer undergoing MTP, feelings of family obligation were associated with their willingness to receive such results, but also that this information was perceived as burdensome and/or inconsequential by patients, given their disease stage [8].

There is concern that MTP may hold psychological risks [9, 10]. It is possible that patients with a cancer diagnosis may hold high hopes for MTP to provide new treatments [11], and feel disappointed if no actionable result is found, even if they had been advised that the chances of a useful outcome were low. Equally, if clinically actionable results are found, and the relevant drug is not available to the patient, or they are deemed too unwell for treatment, they may feel angry and abandoned.

This study aimed to elicit the attitudes and expectations of participants with advanced cancer towards MTP and return of results prior to undergoing testing, to determine what support and information may need to be provided for patients in the clinical setting, specifically at the time of consent.

## Methods

### Participants

The Molecular Screening and Therapeutics (MoST) Program is a study underway at the Garvan Institute of Medical Research in Sydney, Australia [12]. It is recruiting 1,000 adult participants with pathologically confirmed advanced or metastatic solid cancer, with a particular focus on rare cancer. Participants undergo MTP and, if there are actionable findings, are enrolled in a related therapeutic trial if available (including access to immune checkpoint inhibitors). The Psychosocial Issues in Genomics in Oncology (PiGeOn) Project is a longitudinal, mixed methods psychosocial sub-study of MoST, which aims to examine the psychosocial, behavioral and ethical impact of MTP [11, 13]. Participants gave written consent to participate in PiGeOn while giving consent to participate in MoST. This sub-study was approved by the St Vincent’s Hospital Human Research Ethics Committee.

Details of inclusion and exclusion criteria for MoST are reported in full in the MoST protocol paper [12]. In summary, the target population comprises adult participants with pathologically confirmed advanced or metastatic solid cancers of any histological type, either during or after their last line of effective therapy; participants have Eastern Cooperative Oncology Group (ECOG) Performance Status 0, 1 or 2; and have sufficient accessible tissue for molecular profiling. Additional exclusion criteria for the PiGeOn study were inability to comply with study requirements, including timing and/or nature of assessments; and inability to provide written informed consent [13].

### Data collection

Purposive sampling was used to ensure heterogeneity (age, gender, cancer type) in the cohort. Eligible participants were invited to participate in a semi-structured interview immediately after giving written consent for MTP within the MoST Program, and to the PiGeOn study. Consenting participants were interviewed by telephone within 1–2 weeks, at a time of their choosing.

Semi-structured interviews were conducted by one researcher (NB) and continued until data saturation was reached (i.e. no new information after three consecutive interviews) [14]. Participants were asked about their understanding of MTP, what influenced their decision to participate, their attitudes toward testing and return of results, managing uncertainty, and who they thought should be able to access testing (see Additional file 1). The interview questions and follow-up prompts were developed iteratively as required to develop themes identified during the study analysis. Demographic details were collected within the parent study (MoST).

In addition to the interview portion of the PiGeOn study, a survey was administered to the entire cohort (*n* = 898), which included an opportunity to provide free-text responses to the questions: *‘What are the benefits of MTP?’* and *‘What are the drawbacks of MTP?’* Answers were extracted from the surveys and content-analyzed alongside the interview data.

### Analysis

Interviews were audio-recorded, transcribed verbatim, and data was analyzed according to thematic analysis [15]. Using line-by-line coding, multi-disciplinary researchers (n = 8) developed initial codes from six transcripts, which were synthesized into focused codes. This initial thematic map was applied to additional transcripts. Using the constant comparative method, new codes were iteratively generated and applied to the data set. Data collection and analysis occurred concurrently as themes were refined and applied to the findings. Any differences among researchers’ interpretations were resolved through discussion and negotiated consensus. The varied academic backgrounds of the researchers (genetics, medicine, bioethics, psychology) ensured reflexivity [16].

The free-text responses from the survey were tabulated and assessed using content analysis [17], allowing for triangulation of data. Rigor was also derived from iterative discussions and review of the coding process by the researchers until thematic coding was complete.

## Results

Thematic saturation was reached when 20 participants were interviewed. No-one who was approached refused to be interviewed. Interviews were conducted between August 2017 and June 2018 and averaged 26 min in length. The mean age of the participants was 57.1 years, and 45% were female. The average ECOG score at the time of recruitment was 1 and the cancer diagnoses were varied (Table 1).

**Table 1:** Demographics

	Qualitative sample *n* = 20	Quantitative sample (responders) *n* = 569	Quantitative sample (non-responders) *n* = 251
Average Age (years)	57.1 (range 41–77)	54 (range 18–85)	56 (range 19–88)
Female (%)	45	52	47
Average ECOG score at recruitment	1 (range 0–2)	1 (range 0–2)	1 (range 0–2)
Cancer diagnosis (%)			
Genito-urinary	30	17	15
Gastro-intestinal	25	22	24
Lung	10	4	3
Breast	5	5	8
Bone and soft tissue	10	21	21
CNS	5	6	12
Other	15	26	17

Of the cohort that completed the survey (820/898 = 91%), 569 (63%) participants responded to the open-ended question about perceived benefits and/or drawbacks of MTP. This data from the free-text survey responses did not reveal any themes beyond those captured in the interviews. Key themes derived from the open-ended written responses are summarized in Table 2.

**Table 2:** Benefits and drawbacks of molecular tumour profiling (*n* = 569) *

Benefits	Frequency	Drawbacks	Frequency
Access to personalised therapy	328 (42%)	No drawbacks	176 (38%)
Research would help others, contribute to scientific advance	250 (32%)	Coping with negative results and possibly other negative information	94 (20%)
Identify cancer risk for family members	141 (18%)	Discrimination/privacy/insurance fears	45 (10%)
Provides hope and possible cure	34 (4%)	Sceptical of science; possible inconclusive or false results	36 (8%)
Gives me certainty, control	10 (1%)	Responsibility or guilt for genes; fear of bearing bad news to family	33 (7%)
Exhausting all options, ticked all boxes	10 (1%)	Burden of study – travel, surveys, waiting for results	29 (6%)
Testing is easy and non-invasive	2 (0%)	Possibility inaccessibility of drug trials if an actionable result was found, or receiving a placebo drug	28 (6%)
		Other	18 (4%)

● May choose more than one

Three themes were identified, with sub-themes in each category. The three main themes were (1): Obvious agreement to participate (2); The black box; and (3) Survival is the priority.

1. **Obvious agreement to participate**

All participants reported no reservations to giving consent to the MTP testing. In response to a question about why they joined the MoST Program, the predominant response was *‘Why wouldn’t you?’* All participants had been undergoing tests and treatments for some time and saw MTP as a continuation of the experience of having advanced cancer. As one participant replied, *‘I didn’t even give it that much thought, it was just another step in the process I’m going through, because I’ll do anything to try and find something that’s going to help me out. So, yeah, I haven’t – didn’t really think too long or hard about it; I just said, “yeah, I’ll do it.”’ (373, male, age 51–60 years)*

However, there were variations in the rationale given for this immediate agreement to genomic testing, as reflected in the four subthemes: a) Access to therapy; b) Self-identified versus oncologist-identified; c) Altruism, and D) Desperation.

- Access to therapy

As participants were eligible for the MoST Program only after they had exhausted all other treatment options, the majority were aware that this study was their last chance to access further therapy. They felt that there was nothing to lose and much to gain by participating. ‘*Where death is looming every day, you know there’s absolutely nothing to worry about here, it couldn’t get any worse’. (Laughs) (356, male, age 51–60 years)*

The MoST research program was seen as an opportunity to receive tailored therapy, thereby bypassing the burdens of potentially avoidable side effects and financial cost associated with non-targeted (and possibly ineffective) treatment. Further, the benefits for joining the study were seen as broader than the possibility of additional cancer therapy for many participants. Undergoing MTP testing was viewed as a way to contribute to the advancement of science and medicine, with some suggesting that this was a moral obligation for all people with cancer. While some participants perceived their participation in testing as *‘helping to find a cure for cancer’*, others held more modest expectations in hoping that if the study results did not help them, it might help someone else. These benefits were seen to clearly outweigh any drawbacks for MTP. MTP was viewed as a low-risk and positive alternative to other avenues of finding treatment such as online personal genomic testing and surgical biopsy, as it was both free of charge and non-invasive for participants. There was an underlying trust in the treating oncologists who drew their attention to the study, which seemed to increase MTP’s acceptability. *‘What have I got to lose? There was really nothing to weigh up. You know, if I can help somebody else then great, if I can help myself, even better but, um, you know, it’s only time there’s nothing very invasive - some blood and a bit of time’. (356, male, age 51–60 years)*

b) Self-identified versus oncologist-identified

Two pathways for identification of the study were evident. Some participants had learnt about the MoST Program online. Many had heard about dramatic responses to immune checkpoint inhibitors while pro-actively searching for treatment options and clinical trials. These “self-referred” participants were characterised by a higher engagement with study processes and their knowledge of available treatment options. These participants also perceived themselves (or the spouse who drove the engagement with the research program) as caring more about their outcomes than anyone else: *“no-one else cares as much as me”. (265, male, age 41–50 years).*

Others found out about the study through their treating oncologists. This subgroup of participants tended to be more passive and accepting of what information was proffered and were much less interested in the research protocol than the self-referred group. A significant trust in the oncologist’s opinion accompanied this position*, ‘well, for me it was I trusted the doctor. You don’t tell the plumber how to do the plumbing, so you don’t tell the doctor how to do the doctoring.’ (375, female, age 61–70 years)*

c) Altruism

While not a primary motivation for participating in genomic testing, altruism played a part in the considerations of most participants. This altruism was focused on the community in general, or more specifically on family members. Participation was seen as worthwhile, whether or not a good outcome was experienced by the participant, if it meant that someone may benefit from their ‘*terrible*’ cancer experience. The results from the research were also seen to be of benefit to younger generations, either as providing advance knowledge of a possible germline variant, or improved hope for cancer cure through early diagnosis. ‘*Because it’s not – it’s not all about me, mate, it’s about everybody that has this cancer’. (705, male, age 71–80 years)* (see Theme 3 below).

d) Desperation

A subgroup of participants reported a feeling of *‘desperation’* about extending their lives, noting that they were running out of treatment options, as if they were a *‘ticking time-bomb’* with death increasingly close. There was a pressing need to know that every treatment option had been pursued. These participants reported finding it difficult to wait 10 weeks for the results and were willing to pay anything to access more treatment options, even if the chance of a positive outcome was low, since it was considered that any chance was better than no chance. This sense of desperation seemed to increase the anxiety they experienced when waiting for results. Participants considered this anxiety to be worth tolerating, with many noting that all hope would be lost if MTP did not provide an answer*. ‘Well, it’s pretty simple. When you’ve got no other hope or no other opportunity and no other idea what the hell is going on, you grab every chance you can grab.’ (382, male, age 61–70 years)*

2) **The black box**

Participants’ understanding of MTP was generally poor, despite explanations provided during the consent process. MTP was viewed as a *‘black box’*, the workings of which were obscure. This was expressed with regard to three sub-themes: a) Understanding the study, b) The science, and c) Beyond my capacity.

- Understanding the study

Participants were generally cognizant of their lack of understanding, and while some would have liked more information (and sought it online), this did not affect their decision to provide consent. The focus of these participants was on access to new therapy, and they either did not care what the access process involved or were unreservedly accepting of the consent information provided. Any feelings of frustration tended to focus on issues of access to the trial. Participants made comments such as: – ‘*Why was I not told of the MoST Program sooner?’; ‘What would have happened if I had not found the study online?’* and *‘Why did I have to wait until treatment options were exhausted before being eligible?’* Specific concerns were held by the majority of participants who lived in interstate and regional areas, because access issues related to the costs of travel to the hospital and city accommodation were troublesome. This frustration was balanced by another cohort who was overtly appreciative of their opportunity to join the study and feeling lucky they had*. ‘It’s probably not that important to actually know … the science behind it. I don’t really care about* (the details) *as long as* (I get) *the treatment options.’ (340, male, age 41–50 years)*

b) Science

The lack of understanding of the testing process was counter-balanced by a confidence in the ‘*power of science’*. This was either expressed as a general trust in the scientific process, and that any advance would be advantageous, or personalized by the treating oncologists, who were perceived as understanding the process and able to provide relevant information as needed. One participant explained: *‘it’s* (MTP) *more of a doctor thing. I feel I would like a little bit more information on it, now that you mention it, it would be interesting, but, it’s not something I’ve felt the need to ask about … , but, really, until they know what it is, tell me if there’s any treatments, and, I’m quite happy just to leave it in their* (the doctor’s) *hands.’ (276, female, age 51–60 years)*

c) Beyond my capacity

Many participants acknowledged that they did not understand the MoST Program and did not attempt to understand MTP, as they considered genomic testing to be *‘beyond’* them. While these participants were not troubled by their ignorance, it was noted that many had an incorrect understanding of the test implications. This became evident from wrong inferences, such as being concerned that having the test may impact their access to insurance products or confusing somatic with germline results, and thinking that somatic variants would be passed onto family and could influence reproductive decision-making for their children.

‘*I’m weighing up potential benefits (*of MTP) *for my children … the down side of that which is … the potential impact on their lives from the point of view of life insurance policies, employment and all sorts of other things.’ (382, male, age 61–70 years)*

3) **Survival is the priority**

Overall, this group of participants were advanced cancer sufferers striving to stay alive through access to new treatment, with subthemes focussing on: a) Preserving hope; b) Treatment is the priority; and c) Fear the cancer, not the treatment.

- Preserving hope

The MoST Program was perceived as an opportunity to preserve hope for prolonged survival. The participants with this view were still ‘*trying to fight their cancer’*, eager for ongoing investigation which might uncover something helpful. They felt that by joining the MoST Program, they had extra people on their *‘side’, ‘looking out for them’* and helping them. Just being associated with a big hospital was felt to increase opportunities for drug trials. *‘ … getting the right people on side, who are going to help you, and work with you all the way.’ (323, male, age 61–70 years).*

Even if treatment was not available through a study related to the MoST Program, participants envisioned taking their results with them to find treatment elsewhere. Despite being told that the chances of a positive result were small, many participants were confident that *‘something would be found’* to help.

*‘One of the reasons I’m doing the* (MTP) *test is because it may not be something that may happen now or one year or five years or 10 years down the track, but it could.’ (316, male, age 71–80 years).*

b) Treatment is the priority

Finding a new treatment was the overriding motivation for joining the MoST Program for the majority of these participants. Despite the investigators’ concern regarding the psychological impact of genomic testing on them, participants were not very interested in exploring a negative impact for themselves. Non-cancer information (such as secondary findings) was viewed as irrelevant and burdensome at a time when they had *‘more important’* (i.e. existential) things to consider. Many participants seemed to lack emotional resources to countenance disclosing germline findings to family (if these were to be forthcoming), even if they saw it was important. Generally, they said that communication of relevant results to relevant biological relatives would occur if it did not prove to be too difficult.

c) Fear the cancer, not the treatment

All participants were given the opportunity to discuss what they saw as the drawbacks to MTP. It was clear that any fear that existed was a result of progressive disease, which dominated their thoughts, and not MTP. The only ‘fear’ associated with testing was the prospect of a poor survival outcome. Having undergone many tests previously, genomic testing was seen as just another test, no different to previous tests: *‘Oh, no, that’s not the testing, that, that impact is just knowing I’m in stage 4, you know, that, that’s got nothing to do with the testing, which comes and goes, which comes and goes, it’s okay.’ (375, female, age 61–70 years).*

## Discussion

This qualitative study explored the experiences of adults with advanced cancer 2 weeks after agreeing to undergo MTP to potentially identify personalised treatment after all other therapeutic options had been exhausted. This cohort had a strong desire to find new treatments whilst being generally unconcerned about the potential to identify germline variants relevant to family members. Further, although participants did not clearly understand the technical details of what genomic testing involved, participants were happy to trust their oncologist to report relevant information. Some were wary of obtaining a disappointing outcome, but the majority were quite hopeful at the time of interview.

All participants appeared to give consent to the study without reservation, because it was non-invasive, and represented an opportunity for new therapy. Yet while MTP is in many ways *‘just another test’*, any testing of the genome carries the possibility that germline variants may be found which will have implications for both the participant and their blood relatives. Mandelker and colleagues compared tumor and germline sequencing in 1040 patients with advanced cancer and identified clinically actionable inherited pathogenic variants in the ACMG notifiable autosomal dominant cancer genes in 9.6% of the sample [7]. Schrader and colleagues found pathogenic variants in 4.7% of a sample of 1566 patients with advanced cancer [18]. Despite careful explanation at the time of consent, there was a wide variability in understanding of the MTP process and its implications in our cohort. Previous authors have expressed concern about the validity of consent in this setting [19]. While there is an obvious motivation for these patients to undergo testing, there is some concern that the participants are not critically reflecting on the fact that being a MoST participant could provide them with a finding that has implications for their relatives. As cancer patients are inundated with information regarding their disease, including material about treatment and associated tests, mechanisms of consent to MTP need to facilitate patients’ understanding of the test’s potential germline implications.

A number of qualitative participants expressed concern about the impact of participation on access to insurance, a response also found in 10% of the quantitative sample. This finding reflected a misunderstanding both of the nature of results, but also of the basis of insurance policies in Australia. Concern that MTP might impact access to health insurance was baseless in this cohort, firstly for participants (as insurance in Australia is risk-rated only, having advanced cancer means that this cohort would not be able to access new risk-rated products anyway) and most family members (as somatic variants are rarely of germline origin and findings are relevant for the patient only). Furthermore, a moratorium on mandatory revelation of adverse genetic test results for life insurance applications is imminent in Australia [20]. However, in other countries, patients experience legitimate anxiety regarding genetic discrimination and cost-related barriers to care [21]. Regardless of local policy, it is of concern if misunderstanding of the test implications increases patient distress, or discourages potential beneficiaries from participating in MTP protocols [22].

Some participants noted that possible germline implications of testing may be a burden for them in the context of advanced illness. This raises the issue of how to promote the dissemination of this information – particularly information that is actionable - to relevant blood relatives [23–25]. While family members may elect to not investigate further, it is widely accepted that they should be given an opportunity to consider this decision for themselves [26–28]. While clinical practice in this area is still emerging, findings arising from research like ours may be critical in guiding clinical pathways.

While this cohort had little understanding of genomic testing, there is scant evidence that patients generally understand the workings of any medical investigation. Biesecker and colleagues have commented that so long as patients understand the ‘gist’ of genetics that may be sufficient [29]. It also reflects wider ethical considerations of what consent to genomics-based tests should look like; namely a shift towards more generic consent that should not assume a granular understanding [30]. What is more important for consent, and which the current participants themselves desired, was information on how the test would impact on them personally, as has been previously reported [31]. The majority of participants realised that MTP may be used to access personalised treatment, and this level of understanding is commensurate with the level of understanding achieved when participating in clinical trials and accessing medical interventions. It could be that clinicians and researchers are unduly concerned about patients and participant’s understanding of the complexity of genomic testing and its implications, when patients and participants are able to critically engage with the test process without having this kind of understanding. This was further emphasised by participants who focused their concern on their disease progression rather than on any investigation, as the desire to live drove and dominated their decision-making. It has been previously noted that the perspective of the patient differs from that of a well person [32]. This point raises the question of genetic exceptionalism and the degree to which it may influence or assume a role in decision-making around genomic testing.

Further, in our study, we found (unrealistic) optimism and high expectations and hope that an actionable result and an available drug trial would be available and successful, despite being advised of the unlikelihood of this outcome. There is therefore a potential for emotional distress given the low likelihood of an effective targeted treatment. Oncologists may find it difficult to discuss the negative aspects of prognosis with patients [33], which can contribute to these unrealistic hopes [34]. Clinicians may need to learn advanced communication strategies to help patients manage their disappointment in the case of no actionable results [35]. Well accepted evidence-based communication strategies, including patient prompt lists and decision aids, may further facilitate communication in this complex setting [36–39].

This study provides useful participant perspectives on a new form of testing. The strengths of this study is its ecological validity, as participants were about to undergo MTP in a clinical context where it may be implemented in routine care in the near future. Participants were purposively sampled from a large pool, providing a cohort with diverse demographic and disease characteristics. A limitation of this study is that participant recall may have been negatively impacted by the experience of advanced disease.

## Conclusions

This study explored the attitudes of adults with advanced cancer towards genomic testing for personalized therapy. We found that management of expectations may be needed to help patients accept that the chance of a result that would change cancer treatment is low. However, in general, participants were both positive and optimistic about participating in the process of genomic testing, despite their limited understanding of the test and its implications. Our ongoing longitudinal follow-up will elucidate whether this positive view is maintained following receipt of MTP results.

## Additional file

Additional file 1: Interview Schedule. 18 questions which asked of each interviewee. (DOCX 14 kb)

## Data Availability

The datasets generated and/or analysed during the current study will be available from the corresponding author on reasonable request.

## Abbreviations

MoST: Molecular Screening and Therapeutics Program
MTP: Molecular tumor profiling
PiGeOn: Psychosocial Issues in Genomics in Oncology

## References

[1] André F, Bachelot T, Commo F, Campone M, Arnedos M, Dieras V (2014). Comparative genomic hybridisation array and DNA sequencing to direct treatment of metastatic breast cancer: a multicentre, prospective trial (SAFIR01/UNICANCER). Lancet Oncol.

[2] Horak P, Fröhling S, Glimm H (2016). Integrating next-generation sequencing into clinical oncology: strategies, promises and pitfalls. ESMO Open.

[3] Yanes T, Willis AM, Meiser B, Tucker KM, Best M (2018). Psychosocial and behavioral outcomes of genomic testing in cancer: a systematic review. Eur J Hum Genet.

[4] Gray Stacy W., Hicks-Courant Katherine, Lathan Christopher S., Garraway Levi, Park Elyse R., Weeks Jane C. (2012). Attitudes of Patients With Cancer About Personalized Medicine and Somatic Genetic Testing. Journal of Oncology Practice.

[5] Yusuf RA, Rogith D, Hovick SR, Peterson SK, Burton-Chase AM, Fellman BM (2015). Attitudes toward molecular testing for personalized cancer therapy. Cancer.

[6] Blanchette PS, Spreafico A, Miller FA, Chan K, Bytautas J, Kang S (2014). Genomic testing in cancer: patient knowledge, attitudes, and expectations. Cancer.

[7] Mandelker D, Zhang L, Kemel Y (2017). Mutation detection in patients with advanced cancer by universal sequencing of cancer-related genes in tumor and normal dna vs guideline-based germline testing. JAMA.

[8] Miller FA, Hayeems RZ, Bytautas JP, Bedard PL, Ernst S, Hirte H (2014). Testing personalized medicine: patient and physician expectations of next-generation genomic sequencing in late-stage cancer care. Eur J Hum Genet.

[9] Liang R., Meiser B., Smith S., Kasparian N.A., Lewis C.R., Chin M., Long G.V., Ward R., Menzies A.M., Harris-Wai J.N., Kaur R. (2016). Advanced cancer patients’ attitudes towards, and experiences with, screening for somatic mutations in tumours: a qualitative study. European Journal of Cancer Care.

[10] Wakefield CE, Kasparian NA, Meiser B, Homewood J, Kirk J, Tucker K (2007). Attitudes toward genetic testing for cancer risk after genetic counseling and decision support: a qualitative comparison between hereditary cancer types. Genet Test.

[11] Goldberg C. Concerns that rosy direct-to-consumer ads hype Cancer drugs to vulnerable patients 2017. Available from: https://www.wbur.org/commonhealth/2017/01/31/direct-to-consumer-cancer-drugs. [cited 2018 08/11/2018].

[12] Thavaneswaran S, Sebastian L, Ballinger M, Best M, Hess D, Lee CK (2018). Cancer molecular screening and therapeutics (MoST): a framework for multiple, parallel signal-seeking studies of targeted therapies for rare and neglected cancers. Med J Aust.

[13] Best M, Newson AJ, Meiser B, Juraskova I, Goldstein D, Tucker K (2018). The PiGeOn project: protocol for a longitudinal study examining psychosocial, behavioural and ethical issues and outcomes in cancer tumour genomic profiling. BMC Cancer.

[14] Bowen GA (2008). Naturalistic inquiry and the saturation concept: a research note. Qual Res.

[15] Braun V, Clarke V (2006). Using thematic analysis in psychology. Qual Res Psychol.

[16] Berger R (2015). Now I see it, now I don’t: researcher’s position and reflexivity in qualitative research. Qual Res.

[17] Hansen Anders, Cottle Simon, Negrine Ralph, Newbold Chris (1998). Content Analysis. Mass Communication Research Methods.

[18] Schrader KA, Cheng DT, Joseph V (2016). Germline variants in targeted tumor sequencing using matched normal dna. JAMA Oncol.

[19] Sulmasy DP, Astrow AB, He MK, Seils DM, Meropol NJ, Micco E (2010). The culture of faith and hope: patients’ justifications for their high estimations of expected therapeutic benefit when enrolling in early phase oncology trials. Cancer.

[20] FSC moves to prohibit genetic test disclosure. https://www.insurancenews.com.au/life-insurance/fsc-moves-to-prohibit-genetic-test-disclosure. Accessed 9 May 19.

[21] Armstrong K, Weber B, FitzGerald G, Hershey JC, Pauly MV, Lemaire J (2003). Life insurance and breast cancer risk assessment: adverse selection, genetic testing decisions, and discrimination. Am J Med Genet A.

[22] Armstrong K, Putt M, Halbert CH, Grande D, Schwartz JS, Liao K (2012). The influence of health care policies and health care system distrust on willingness to undergo genetic testing. Med Care.

[23] Chivers Seymour K, Addington-Hall J, Lucassen AM, Foster CL (2010). What facilitates or impedes family communication following genetic testing for Cancer risk? A systematic review and meta-synthesis of primary qualitative research. J Genet Couns.

[24] Gaff CL, Collins V, Symes T, Halliday J (2005). Facilitating family communication about predictive genetic testing: Probands’ perceptions. J Genet Couns.

[25] Metcalfe A, Coad J, Plumridge GM, Gill P, Farndon P (2008). Family communication between children and their parents about inherited genetic conditions: a meta-synthesis of the research. Eur J Hum Genet.

[26] Roshanai AH, Lampic C, Rosenquist R, Nordin K (2010). Disclosing cancer genetic information within families: perspectives of counselees and their at-risk relatives. Familial Cancer.

[27] Forrest K, Simpson S, Wilson B, Van Teijlingen E, McKee L, Haites N (2003). To tell or not to tell: barriers and facilitators in family communication about genetic risk. Clin Genet.

[28] Morrow A, Jacobs C, Best M, Greening S, Tucker K (2018). Genetics in palliative oncology: a missing agenda? A review of the literature and future directions. Support Care Cancer.

[29] Biesecker B, Austin J, Caleshu C (2017). Theories for psychotherapeutic genetic counseling: fuzzy trace theory and cognitive behavior theory. J Genet Couns.

[30] Ayuso C, Millán JM, Mancheño M, Dal-Ré R (2013). Informed consent for whole-genome sequencing studies in the clinical setting. Proposed recommendations on essential content and process. Eur J Hum Genet.

[31] Hammami MM, Al-Jawarneh Y, Hammami MB, Al Qadire M (2014). Information disclosure in clinical informed consent:“reasonable” patient’s perception of norm in high-context communication culture. BMC Med Ethics.

[32] Matsuyama R, Reddy S, Smith TJ (2006). Why do patients choose chemotherapy near the end of life? A review of the perspective of those facing death from cancer. J Clin Oncol.

[33] Hancock K, Clayton JM, Parker SM, Wal d S, Butow PN, Carrick S (2007). Truth-telling in discussing prognosis in advanced life-limiting illnesses: a systematic review. Palliat Med.

[34] DeMartini J, Fenton JJ, Epstein R, Duberstein P, Cipri C, Tancredi D (2018). Patients’ hopes for advanced Cancer treatment. J Pain Symptom Manag.

[35] Temel JS, Gainor JF, Sullivan RJ, Greer JA (2018). Keeping expectations in check with immune checkpoint inhibitors. J Clin Oncol.

[36] Campbell TC, Carey EC, Jackson VA, Saraiya B, Yang HB, Back AL (2010). Discussing prognosis: balancing hope and realism. Cancer J.

[37] Miller N, Rogers S (2018). A review of question prompt lists used in the oncology setting with comparison to the patient concerns inventory. Eur J Cancer Care.

[38] Kiely BE, Tattersall MH, Stockler MR (2010). Certain death in uncertain time: informing hope by quantifying a best case scenario. J Clin Oncol.

[39] Kiely B, McCaughan G, Christodoulou S, Beale P, Grimison P, Trotman J (2013). Using scenarios to explain life expectancy in advanced cancer: attitudes of people with a cancer experience. Support Care Cancer.

