# Supplementary material for: Patient perspectives on molecular tumor profiling: “Why wouldn’t you?”
Source: BMC Cancer. 2019 Jul 31;19:753. doi: 10.1186/s12885-019-5920-x (PMC6670204; doi:10.1186/s12885-019-5920-x)
Supplement: Supplementary file 1 — Interview Schedule. 18 questions which asked of each interviewee. (DOCX 14 kb) [file 12885_2019_5920_MOESM1_ESM.docx]

**Supplementary File 1: Interview Schedule***.*

1. Tell me a bit about yourself. How did you come to know about the study?
2. Can you tell me what you know about the blood test you had for the study, if anything?
3. Had you heard of tumour genetic panel testing before the study?
4. Why do you think you were offered the opportunity to be in this study?
5. What do you think about genetic panel testing?
6. What do you see as the benefits (if any) of genetic panel testing?
7. Is there anything that worries you about it?
8. Do you think genetic panel testing should be offered to people like you?
9. How do you think we should decide who gets these tests, if a public healthcare system can’t fund them for everyone?
10. Before deciding to have this test, do you think people should be provided with some resources or information? If so, what – and how? How long do you think they should have to decide about the test before it takes place? What process do you think people should go through before deciding about this test?
11. I understand you are currently waiting for your test to be done. How are you feeling about this?
12. Do you think you should receive all the information found in your test, or just the information relevant to your treatment?
13. How do you think you would feel if you were told that changes in your genes had been found, but that the meaning of this change was uncertain or could not be used to inform your treatment?
14. What do you think makes genetic information valuable to people?
15. Have you thought about whether this blood test would be of interest to your relatives? Do you plan to talk to your relatives about your test and the results?
16. Do you think it’s okay to participate in this kind of research but choose not to receive any results at all? Or is it okay for people to be given genetic information, say if it could lead to cancer treatment, even if they said they didn’t want to receive it?
17. How do you generally deal with uncertainty in your life?
18. Is there anything else you would like to say about genetic panel testing? Do you have any advice about how this topic should be discussed with other patients?
